# Observational study of the relationship between negative cognitive processing bias and mental health, sleep quality in the early and middle stages of peacekeeping mission

**DOI:** 10.1097/MD.0000000000042295

**Published:** 2025-05-02

**Authors:** Wen Jiang, Yiyi Zhang, Xu Dong, Kaiwen Hou

**Affiliations:** a Outpatient Department, The General Hospital of Western Theater Command, Chengdu, Sichuan Province, China.

**Keywords:** cognition, mental health, military, peacekeeping, sleep quality

## Abstract

To examine the correlation between negative cognitive processing bias and the mental health, sleep status of peacekeepers across various time periods to establish a benchmark for peacekeeper mental health interventions. Symptom checklist 90 (SCL-90), Pittsburgh sleep quality index (PSQI), and negative cognitive processing bias questionnaire (NCPBQ) were adopted to investigate 172 Chinese peacekeepers in the Democratic Republic of the Congo at the early and middle stages of the mission. There was no statistically significant difference in the overall score of PSQI between the early and middle stages of the mission (*P* = .699). However, there was a statistically significant difference in the overall score of SCL-90 and NCPBQ (*P* < .05). Furthermore, there was a positive correlation between the negative cognitive processing bias and the scores of SCL-90 and PSQI (*R* = 0.114–0.528, *P* < .05). A negative cognitive processing bias was also found to be a strong predictor of the overall score on the SCL-90 and PSQI assessments, with explanation rates of 27.3% and 17.5%, respectively. Peacekeepers are prone to experiencing psychological issues during the initial phase of their mission, necessitating careful attention. The presence of negative cognitive processing bias significantly impacts both mental health and sleep quality. Alleviating negative cognitive processing bias can potentially enhance the mental well-being and sleep quality of peacekeepers.

## 1. Introduction

The primary objectives of the United Nations (UN) peacekeeping missions are to prevent hostility, restore stability, and maintain peace in conflict-ridden areas.^[1]^ The environments where peace operations deploy are stressful and emotionally straining, and witnessing the effects of war and atrocities can impact the mental health of any individual. The current focus is on addressing mental health concerns in peace operations, including the prevalence of stress and anxiety, depression, and other mental disorders among peacekeepers.^[2]^

Peacekeeping operations involve varying stressors and demands between operations and within an operation.^[3]^ One can roughly categorize the stressors as daily difficulties or high-magnitude stressors. Daily difficulties include poor living conditions, a narrow range of the camp, strict military management, repetitive work content, and homesickness. High-magnitude stressors include volatile regional security situations, frequent armed conflicts, sudden violence, and multiple deployments. These repeated exposure to combat and other stressors are all associated with mental health issues.^[4]^ Furthermore, COVID-19 has spread at an unprecedented speed worldwide since its outbreak in December 2019. The number of COVID-19 cases is also escalating rapidly in Africa, where response capacity has been assessed as substantially limited due to a lack of medical resources.^[5]^ Combined with the sudden outbreak of other infectious diseases, such as malaria and Ebola, it has brought more pressure on the personnel performing the UN Stabilization Mission in the Democratic Republic of the Congo (MONUSCO).

Another key point that causes mental health problems for peacekeepers is the cognitive style. Cognitive processing has a moderating role in the relationship between stressor exposure or other risk factors on the one hand and psychopathology or other undesired outcomes on the other hand.^[6]^ Negative cognitive processing bias is a cognitive trait that directs attention to negative internal or external stimuli and leads to misinterpretation of this information more negatively.^[7]^ From the survival perspective, negative cognitive processing bias is a self-protection mechanism of individuals and a kind of automatic processing formed by individuals in long-term evolution.^[8]^ However, excessive negative cognitive processing bias may affect mental health and even lead to mental disorders. In this context, negative cognitive biases in self-referential processing, attention, interpretation, and memory play a crucial role in the use of maladaptive emotion regulation strategies such as increasing rumination, decreasing distraction, and reappraisal; these strategies further intensify and perpetuate symptoms associated with depression.^[9]^ Traditional wisdom holds that ruminating is a maladaptive strategy for regulating emotions; it consists of passively and repetitively contemplating negative mood states or their causes and effects. Negative memory bias exhibits mood-congruent memory biases, evidenced by preferential recall for negative information. And negative attention bias acts as the first filter for information selection and shows an attentional preference for negative stimuli and deviation from positive stimuli.^[10]^

Peacekeepers experience a significant prevalence of mental health issues due to the accumulation of many stressors and cognitive risk factors. Based on the UN investigation, over half of the participants reported experiencing symptoms that align with a mental health disorder. This proportion is notably more than that observed in the whole population. 49% of participants exhibited indications of at least 1 mental health condition, whereas 22% displayed indications of 2 or more. Approximately 18% of participants were identified as having generalized anxiety disorder, whereas 23% were marked for major depressive illness.^[11]^ The prevalence of sleeplessness among young and middle-aged peacekeepers is as high as 9.3%.^[12]^ Inadequate sleep can exacerbate the intensity of psychological symptoms, such as depression.^[11]^ And due to high levels of exposure to potentially traumatic events on deployment, such as being threatened with injury or death, seeing dead bodies, witnessing degradation and misery, and hearing of a friend or coworker being injured or killed, peacekeepers had significantly high prevalence of post-traumatic stress disorder as 16.8%.^[13]^

Addressing the mental health concerns of peacekeepers is imperative, as it will not only enhance their performance but also foster a more compassionate and humane environment inside the UN. This study examines the mental well-being of Chinese peacekeeping officers and soldiers stationed in the Democratic Republic of the Congo (DRC). It seeks to identify any changes in their mental health and sleep quality, as well as understand the characteristics of their cognitive processing patterns and how they impact their mental well-being. The findings of this study will serve as a reference for future mental health initiatives and the development of effective psychological intervention strategies.

## 2. Method

### 2.1. *Subjects*

All participants were Chinese military personnel employed by the People’s Liberation Army (PLA). The sample consisted of 175 soldiers and officers deployed for 12 months from October 2021 to October 2022 in DR Congo, serving in the MONUSCO engineer unit. Of the invited personnel, 172 (98.29%) completed the survey. Only 3 participants did not complete the study (for organizational reasons). This study was approved by the ethics committee of the General Hospital of Western Theater Command (approval number: CHL20210701). Written informed consent was obtained from individual participants. Table S1 reports the demographic characteristics of the respondents.

**Table 1:** Correlation between negative cognitive processing bias and SCL-90 in peacekeepers (*r*).

Variable	Negative attention bias	Negative memory bias	Rumination	NCPBQ total score	Somatization	Compulsive symptom	Interpersonal sensitivity	Depression	Anxiety	Hostility	Phobia	Paranoid	Psychotic symptom	Sleep and eating problems	SCL-90 total score
Negative attention bias	1														
Negative memory bias	0.814**	1													
Rumination	0.805**	0.746**	1												
NCPBQ Total score	0.945**	0.943**	0.883**	1											
Somatization	0.468**	0.457**	0.513**	0.508**	1										
Compulsive symptom	0.491**	0.511**	0.508**	0.540**	0.886**	1									
Interpersonal sensitivity	0.481**	0.44**	0.521**	0.507**	0.834**	0.878**	1								
Depression	0.476**	0.432**	0.516**	0.501**	0.878**	0.903**	0.934**	1							
Anxiety	0.477**	0.44**	0.505**	0.502**	0.881**	0.901**	0.949**	0.965**	1						
Hostility	0.472**	0.42**	0.495**	0.489**	0.874**	0.89**	0.941**	0.965**	0.961**	1					
Phobia	0.453**	0.383**	0.475**	0.460**	0.833**	0.830**	0.928**	0.938**	0.953**	0.934**	1				
Paranoid	0.469**	0.42**	0.483**	0.485**	0.842**	0.878**	0.958**	0.957**	0.961**	0.954**	0.956**	1			
Psychotic symptom	0.462**	0.403**	0.505**	0.479**	0.849**	0.872**	0.954**	0.96**	0.965**	0.948**	0.965**	0.968**	1		
Sleep and eating problems	0.473**	0.469**	0.498**	0.512**	0.866**	0.892**	0.859**	0.909**	0.896**	0.892**	0.840**	0.861**	0.858**	1	
SCL-90 Total score	0.495**	0.462**	0.528**	0.524**	0.923**	0.939**	0.96**	0.982**	0.982**	0.974**	0.951**	0.969**	0.971**	0.928**	1

NCPBQ = negative cognitive processing bias questionnaire, SCL-90 = symptom checklist 90.

** *P* < .01.

### 2.2. *Design*

We used a longitudinal repeated measures design. The participants were measured at 2 time points (T1, T2): the first month during deployment (T1) and the fourth month during deployment (T2). Participation was voluntary, and written consent was obtained from all participants. Data collection was conducted in the presence of an on-site researcher, who was a psychologist from the medical unit of the same batch of peacekeeping missions. And the questionnaires were hetero-administered. Due to changes in the commander’s deployment arrangements, no approval was given for data collection later in the peacekeeping mission.

Statistical analyses were performed using SPSS (IBM Corp.; released in 2020; IBM SPSS Statistics for Win; Version 27.0; Armonk: IBM Corp). Based on the experimental data, descriptive analysis, normality test, paired sample *t*-test, Pearson correlation analysis, and regression analysis performed with *P* < .05, which was considered statistically significant. Data from the 2 time points were combined for correlation analysis and regression analysis. The regression analysis of the total score was performed by input linear regression, and the regression analysis of each factor was performed by stepwise linear regression.

## 2.3. *Measures*

### 2.3.1. *Symptom checklist 90 scale.*

 This questionnaire contains a wide range of psychiatric symptomatology. It includes 90 items, dividing into 10 factors: somatization, obsessive-compulsive symptoms, interpersonal sensitivity, depression, anxiety, hostility, phobic anxiety, paranoid ideation, psychoticism, and others (sleep and eating problems). Each item is graded from 1 to 5 according to the severity (from none to extreme). The symptoms were considered affirmative when the total score exceeded 160 points or a factor score of ≥ 2 (factor score = total score of all items contributing to the factor/number of items contributing to the factor). A higher score indicated more serious psychiatric symptoms and issues. The present study used the Chinese version of SCL-90. The validity of the total scale was 0.97 in the Chinese population.^[14,15]^ Cronbach α of the present study was 0.99.

### 2.3.2. *Pittsburgh Sleep Quality Index.*

 This questionnaire allows for the evaluation of the sleep quality over 1 month. It comprises 7 components: sleep quality, sleep latency (the time it takes to fall asleep), sleep duration (the length of time spent sleeping), sleep efficiency (the effectiveness of sleep about time spent in bed), sleep disorders, medication use to sleep, and daytime sleepiness (the level of tiredness experienced during the day). Through the calculation of the rules, each component is scored according to 0 to 3 points, and the cumulative score of each component is the total score of PSQI. The total score ranged from 0 to 21, and the higher the score, the worse the sleep quality. The cutoff point for determining sleep quality is PSQI ≤ 5 for excellent sleep quality and PSQI > 5 for poor sleep quality.^[16]^ The overall Cronbach alpha coefficient was 0.85 in the Chinese population.^[17]^ In the present study, Cronbach α was 0.798.

### 2.3.3. *Negative Cognitive Processing Bias Questionnaire.*

 The questionnaire is used to evaluate the processing preference for negative information in the cognitive trait. It contains 16 items, dividing into 3 dimensions: negative attention bias, negative memory bias, and rumination. Each item is graded from 1 to 4 according to the suitability (from “not match” to ‘perfect match’).^[18]^ The total score is the sum of each item. The higher the score, the more obvious the negative cognitive processing bias. The overall Cronbach alpha coefficient was 0.89 in the Chinese college students. In this analysis, Cronbach α was 0.88.

## 3. Results

### 3.1. *Demographics*

Table S1, Supplemental Digital Content (https://links.lww.com/MD/O764), depicts the demographic characteristics of the sample. All participants were male (100%), aged 20 to 47 (average: 26.72 ± 4.27) years old, with 3 to 30 (8.83 ± 4.52) years of military service. The majority were single privates. More than two-thirds of participants were below a bachelor degree.

### 3.2. *SCL-90 results*

**Figure 1.**
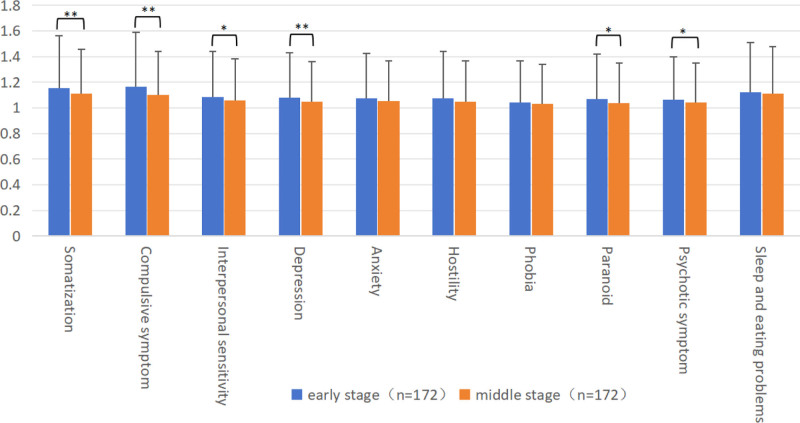
Comparison of SCL-90 results of participants in the early and middle stages of peacekeeping mission. Paired *t*-test was used to compare the mean and standard deviation of the SCL-90 factor scores of participants in the early and middle stages of the peacekeeping mission, including somatization, compulsive symptom, interpersonal sensitivity, depression, anxiety, hostility, phobia, paranoid, psychotic symptom, sleep and eating problems. Pairwise comparison showed that the factor scores of somatization, compulsive symptom, interpersonal sensitivity, depression, paranoid and psychotic symptoms were significantly higher in the early stage than those in the middle stage of the mission. * *P* < .05, ** *P* < .01. SCL-90 = symptom checklist 90.

Participants in the early and middle portions of the peacekeeping deployment exhibited notable disparities in both the SCL-90 total score and average positive factors of participants in the early and middle stages of the peacekeeping mission (*P* < .05). Pairwise comparison showed that the total score, average positive factors of SCL-90, and the factor scores of somatization, compulsive symptom, interpersonal sensitivity, depression, paranoid and psychotic symptoms were significantly higher in the early stage than those in the middle stage of the mission (Table S2, Supplemental Digital Content, https://links.lww.com/MD/O764; Fig. 1).

### 3.3. *Sleep quality*

**Figure 2.**
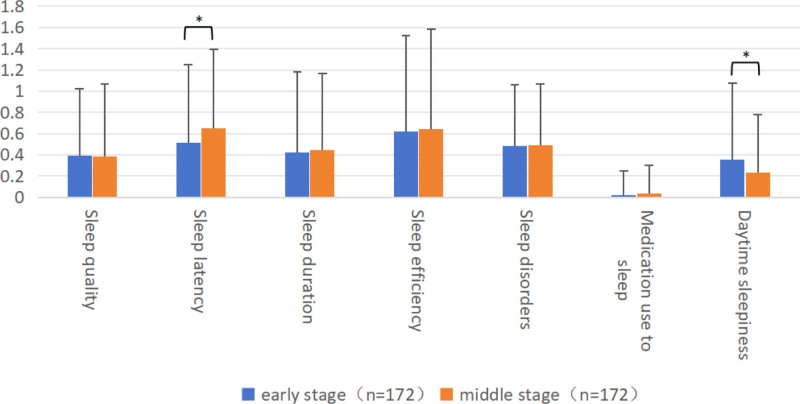
Comparison of PSQI results of participants in the early and middle stages of peacekeeping mission. Paired *t*-test was used to compare the mean and standard deviation of the PSQI factor scores of participants in the early and middle stages of the peacekeeping mission, including sleep quality, sleep latency, sleep duration, sleep efficiency, sleep disorders, medication use to sleep, daytime sleepiness. Pairwise comparison revealed a substantial increase in sleep latency scores during the middle stage compared to the early stage of the mission. And the daytime sleepiness score was higher during the early stage compared to the middle stage of the mission. * *P* < .05. PSQI = Pittsburgh sleep quality index.

Participants in the early and middle stages of the peacekeeping operation did not show a significant difference in the PSQI total score (*P* < .699). However, there were significant variations in sleep latency and daytime sleepiness (*P* < .05). A pairwise comparison revealed a substantial increase in sleep latency scores during the middle stage compared to the early stage of the mission. Additionally, the daytime sleepiness score was higher during the early stage compared to the middle stage of the operation (Table S3, Supplemental Digital Content, https://links.lww.com/MD/O764; Fig. 2).

### 3.4. *Negative cognitive processing bias*

**Figure 3.**
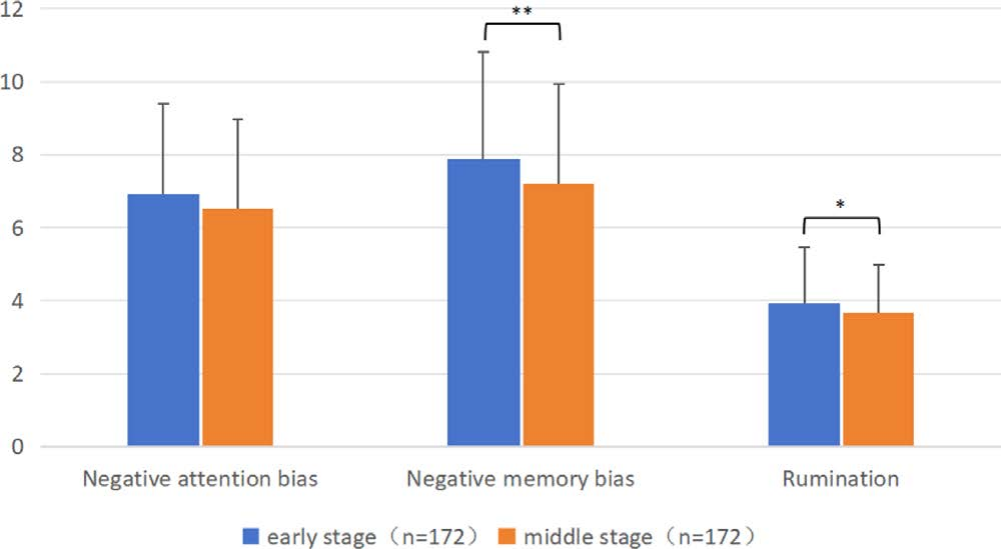
Comparison of NCPBQ results of participants in the early and middle stages of peacekeeping mission. Paired t-test was used to compare the mean and standard deviation of the NCPBQ factor scores of participants in the early and middle stages of the peacekeeping mission, including negative attention bias, negative memory bias, rumination. Pairwise comparison showed that the negative memory bias, and rumination in the early stage were considerably greater than those in the middle stage of the mission. ** *P* < .01, * *P* < .05. NCPBQ = negative cognitive processing bias questionnaire.

Participants in the early and middle stages of the peacekeeping mission had a significant disparity in their NCPBQ total score. Notably, there were significant differences in the negative memory bias and rumination (*P* < .05). Pairwise comparison showed that the total score of NCPBQ, the negative memory bias, and rumination in the early stage were considerably greater than those in the middle stage of the mission (Table S4, Supplemental Digital Content, https://links.lww.com/MD/O764; Fig. 3).

### 3.5. *Correlation analysis*

The data of the 3 scales satisfied the normal distribution. Participants reported positive correlations (*R* = 0.383–0.528, *P* < .01) between the total score of SCL-90 and the following symptoms: somatization, compulsive symptom, interpersonal sensitivity, depression, anxiety, hostility, phobia, paranoid, psychotic symptom, sleep and eating problems. The total score of the NCPBQ was also positively correlated with rumination (Table 1).

The total score of PSQI and the scores of sleep quality, sleep latency, sleep duration, sleep efficiency, sleep disorders, medication use to sleep, and daytime sleepiness of participants showed a positive correlation with the total score of NCPBQ, negative attention bias, negative memory bias and rumination (*R* = 0.114–0.422, *P* < .01) (Table 2).

**Table 2:** Correlation between negative cognitive processing bias and PSQI in peacekeepers (*r*).

Variable	Negative attention bias	Negative memory bias	Rumination	NCPBQ total score	Sleep quality	Sleep latency	Sleep duration	Sleep efficiency	Sleep disorders	Medication used to sleep	Daytime sleepiness	PSQI total score
Negative attention bias	1											
Negative memory bias	0.814**	1										
Rumination	0.805**	0.746**	1									
NCPBQ Total score	0.945**	0.943**	0.883**	1								
Sleep quality	0.321**	0.381**	0.333**	0.374**	1							
Sleep latency	0.346**	0.351**	0.343**	0.373**	0.587**	1						
Sleep duration	0.159**	0.124*	0.214**	0.167**	0.425**	0.339**	1					
Sleep efficiency	0.141**	0.114*	0.168**	0.146**	0.274**	0.271**	0.728**	1				
Sleep disorders	0.31**	0.319**	0.308**	0.336**	0.521**	0.491**	0.245**	0.182**	1			
Medication used to sleep	0.406**	0.35**	0.409**	0.412**	0.368**	0.3**	0.339**	0.21**	0.382**	1		
Daytime sleepiness	0.378**	0.412**	0.376**	0.421**	0.66**	0.454**	0.351**	0.317**	0.51**	0.339**	1	
PSQI total score	0.387**	0.389**	0.409**	0.422**	0.776**	0.713**	0.749**	0.689**	0.644**	0.499**	0.734**	1

NCPBQ = negative cognitive processing bias questionnaire, SCL-90 = symptom checklist 90.

* *P* < .05.

**P < .01.

### 3.6. *Regression analysis*

The linear regression analysis on total score revealed a significant predictive relationship between the NCPBQ total score (independent variable) and the SCL-90 total score (dependent variable). Specifically, the negative cognitive processing bias was found to be a significant predictor of the SCL-90 total score, with an explanation rate of 27.3% (*P* < .001). The stepwise regression analysis on the factors revealed that negative memory bias, when considered alongside negative attention bias and rumination as independent factors, did not significantly impact the total score of SCL-90. Therefore, negative memory bias could not predict the total score of SCL-90. Rumination and negative attention bias could positively predict the total score of SCL-90, with an explanation rate of 28.8% (*P* < .001), as shown in Table 3.

**Table 3:** Regression analysis of negative cognitive processing bias to SCL-90 total score in peacekeepers.

	*B*	β	*t*	*P*	*F*	Adjusted *R*^2^-value
NCPBQ total score	2.493	0.524	11.382	<.001	129.551***	0.273
Rumination	7.631	0.368	4.789	<.001	70.430***	0.288
Negative attention bias	2.404	0.198	2.579	.01		

NCPBQ = negative cognitive processing bias questionnaire, PSQI = Pittsburgh sleep quality index, SCL-90 = symptom checklist 90.

***P < .001.

The linear regression results of the NCPBQ total score as independent variable and the PSQI total score as dependent variable showed that negative cognitive processing bias could significantly predict the total score of PSQI, with an explanation rate of 17.5% (*P* < .001). The stepwise regression analysis on the factors revealed that negative attention bias, negative memory bias, and rumination were used as independent variables to predict the overall score of PSQI. However, the analysis showed that negative attention bias did not significantly impact the total score of PSQI and hence could not be used as a predictor. Rumination and negative memory bias could positively predict the total score of PSQI, with an explanation rate of 17.8% (*P* < .001), as shown in Table 4.

**Table 4:** Regression analysis of negative cognitive processing bias to PSQI total score in peacekeepers.

	*B*	β	*t*	*P*	*F*	Adjusted *R*^2^-value
NCPBQ total score	0.214	0.422	8.603	<.001	74.007***	0.175
Rumination	0.592	0.268	3.639	<.001	38.240***	0.178
Negative memory bias	0.213	0.189	2.576	.01		

NCPBQ = negative cognitive processing bias questionnaire, PSQI = Pittsburgh sleep quality index, SCL-90 = symptom checklist 90.

***P < .001.

## 4. Discussion

Each peace operation and its respective team within a peace operation poses a unique set of challenges for peacekeepers. The levels of risks and insecurity, the quality of infrastructure, the availability of staple supplies, and health hazards vary from 1 UN base to another. The local populace in certain regions of the DRC has stoned or set fire to UN compounds, vehicles, and houses. The attacks, criminality, gunshots, and spontaneous riots are daily features of mission life.^[2]^ Peacekeepers also need to face threats from infectious risks such as malaria and Ebola. The COVID-19 pandemic has exacerbated the health threat. As a result of the travel bans and mobility restrictions imposed to prevent the spread of the virus, personnel feel even more isolated, and the level of support provided to hardship duty stations has been diminished. Although various professional preparations and targeted training had been conducted during the standby training period, those factors put the peacekeepers under great psychological pressure, resulting in a perpetually fluctuating mental state. Moreover, due to the work of the engineer unit being mainly related to construction, only male peacekeepers are deployed. When dealing with stress, men have a smaller support network and lower quality of social support than women.^[19,20]^ The dominant masculinity norms in traditional culture encourage men to become self-reliant and prevent feminine traits such as being weak and emotional. This can lead to denial of symptoms and reluctance to seek medical care, especially for depression and anxiety disorders.^[21,22]^ Therefore, it is necessary to investigate their mental health status.

The survey results showed that the overall mental health level and sleep quality of peacekeepers in the middle stage were better than those in the early stage of the mission, and their cognitive styles also improved. The SCL-90 score in the middle of the mission was significantly lower than in the early stage, especially the scores of somatization, compulsive symptom, interpersonal sensitivity, depression, paranoid, and psychotic symptoms were reduced in the middle stage. However, their overall level of sleep quality did not change significantly; only the time to fall asleep increased in the middle stage, while daytime function improved simultaneously. Regarding negative cognitive processing bias, the scores in the middle stage were also significantly lower than those in the early stage of the task, especially the negative memory bias and negative rumination. Previous research showed that the psychological stress scores of peacekeepers indicate significant stage differences. The proportion of psychological stress in the middle stage is significantly lower than that in the early stage, consistent with the results of this study.^[23]^ One possible explanation is that the initial stress score was elevated due to anticipatory stress.^[3]^ In the early stage, the peacekeepers faced many difficulties; some were already known in the early training, and some were faced after arriving at the mission area. A discrepancy between expectations and reality creates disappointment, obvious negative cognitive processing bias, and a diminished capacity to cope with additional stressors. The peacekeepers might experience less negative stress during the middle stage because of a greater feeling of control over their situation and stressors. Excluding combat-related stressors, there are still numerous stressors in everyday challenges, which can be counterbalanced or surpassed by daily sources of positivity.^[24]^ This process, perceived as positive compared to difficulties, empowers the individual and improves cognitive style. There might also be stress-mitigating properties of the “camp life” that might exceed the stress from the workload and other deployment-related stressors sufficient to attain a positive result. Lifestyle factors (e.g., smoking, surfing the Internet) are associated with sleep quality.^[14]^ The increase in the time to fall asleep might be due to improved network facilities in the middle stage, leading to increased time spent using electronic devices before bed, and more frequent smoking after material replenishment.

The results of the correlation analysis indicated that there was a significant positive relationship between the total and factor scores of the SCL-90 and PSQI and the 3 dimensions of negative cognitive processing bias – negative attention bias, negative memory bias, and negative contemplation bias. This suggests that there is a close association between negative cognitive processing bias and both mental health level and sleep status. Numerous studies have shown a robust link between negative cognitive processing bias and cognitive-related mental health problems.^[25]^ The correlations between negative attention bias and compulsive symptom (*R* = 0.491), negative memory bias and compulsive symptom (*R* = 0.511), and rumination and interpersonal sensitivity (*R* = 0.521) were the strongest among the dimensions of negative processing bias at the level of mental health problems. Negative cognitive processing bias plays a partial mediating role in the relationship between negative life events and obsessive-compulsive symptoms; cognitive bias modification for interpretation and attention can improve obsessive symptoms.^[26]^ Negative cognitive models will affect the individual’s choices of thinking, language, and behavior and affect the evaluation and attribution of self and others, ultimately affecting interpersonal relationships and communication skills. The previous study showed that interpersonal sensitivity is closely related to negative core beliefs about the self, and core beliefs of negative self strongly predicted the main components of interpersonal sensitivity.^[27]^ The strongest correlations in the dimensions of negative processing bias were observed between negative attention bias and medication use to sleep (*R* = 0.406), negative memory bias and daytime sleepiness (*R* = 0.412), rumination and medication use to sleep (*R* = 0.409). Research has demonstrated that a negative cognitive processing bias mediates between neuroticism and the quality of sleep.^[28]^ It is a crucial factor affecting the persistence of sleep disorders.^[29]^ Negative cognition and insomnia also influence each other, forming a vicious circle. Good quality and amount of sleep are fundamental to preserve cognition and affect. Poor quality and short sleep increased negative affect (i.e., anger, fear, and perceived stress) and reduced life satisfaction and positive emotionality.^[30]^

Regression analysis showed that the total score of negative cognitive processing bias had a substantial predictive effect on mental health and sleep quality, the explanation rates were 27.3% and 17.5% respectively. In the stepwise regression analysis for factors, the standard regression coefficient of rumination for predicting mental health level was 0.368, and that for predicting sleep status was 0.268, might be the most important factor. Rumination is closely related to excessive negative emotions, poor problem-solving ability, impaired behavioral activation and inhibition functions, and confusion of attention and consciousness, which play an important role in the occurrence and development of depression. However, this mission is during the COVID-19 epidemic. The study found the continued impact of COVID-related rumination on mental health outcomes throughout the pandemic, including alarmingly high rates of clinically significant levels of anxiety, depression, exhaustion, as well as decreased vigor.^[31]^ Regarding sleep status, according to the cognitive models of insomnia proposed by Espie,^[32]^ dysfunctional cognition may play an important role in perpetuating insomnia; rumination prevents individuals from achieving or maintaining sleep. This theoretical model has also been verified in Tousignant study implying that rumination experienced by individuals before falling asleep will make individuals experience a high degree of cognitive arousal, thus affecting the quality of sleep. Furthermore, when individuals experience intense stress events, rumination could positively predict sleep latency, and the longer the latency is, the worse the sleep quality is. Furthermore, negative attention bias could positively predict the total score of SCL-90 with the standard regression coefficient of 0.198. Increasing evidence indicates that negative attention bias is not solely a phenomenon or symptom associated with some psychological diseases but also a fundamental cognitive factor in their development, maintenance, and recurrence. Attention bias to negative stimuli could result in mental health problems such as anxiety and depression.^[33]^ Further, negative memory bias could positively predict the total score of PSQI with the standard regression coefficient of 0.189. Previous studies have found that poor sleep quality is related to greater depressive symptoms, anxiety, and mood disturbances, which was associated with better memory for negative stimuli and a deficit in sustained attention to non-emotional stimuli.^[34]^

Moreover, the excessive negative processing could be alleviated through cognitive interventions to reduce the adverse effects on mental health. A recent meta-analysis has identified various interventions that can reliably reduce rumination, in particular, approaches that encourage individuals to challenge their thinking style, disengage from the emotional response of rumination, and mindfulness-based approaches.^[35]^ Due to the unique characteristics of peacekeeping deployments, it is particularly important to offer remote services to guarantee the long-term effectiveness of psychological intervention. Adopting a professional and courteous online demeanor, known as a “website” manner, will enhance the effectiveness of the intervention.^[36]^

Nevertheless, the present study has several limitations. First, the sample size was small and the source was relatively single. They were all males from the same unit. Peacekeeping tasks also include supervising ceasefires, observing and reporting on situations, helping to implement peace agreements, preventing illegal border crossings and maintaining security in conflict areas. And gender plays an important role in the expression of endophenotypes (psychophysiological and neuropsychological). The particularity of its task content and gender factors might cause bias in the research results. Thus, these conclusions need to be confirmed in a larger sample. Moreover, due to the limitations of deployment arrangements, this study only conducted surveys in the early and middle stages of the mission, and failed to conduct research in the late stage of the mission, making the research timeline incomplete. In future research, research planning should be carried out before the start of the mission, and the investigation should be carried out throughout the mission period to clarify the changing trend of mental health levels during the mission. Furthermore, mental health and sleep are affected by many factors, not only cognitive style, but also environmental factors such as local conflicts and seasonal changes. PTSD is also a psychological problem with a high incidence rate, but this study included few influencing factors, and no separate investigation was conducted on PTSD or adjustment disorders. Hence, the conclusions of this study can only be used as one of the explanations, further research needs to include more influencing factors and outcome indicators, and cognitive training could be used as an intervention to verify the results of this study.

## 5. Conclusions

In conclusion, peacekeepers are more likely to have negative cognitive bias and a decline in mental health level and sleep status in the early stage of the peacekeeping mission, which is the stage that needs to be paid more attention to. Negative cognitive processing bias is closely related to sleep status and mental health level, especially rumination, which can effectively predict the changes in sleep status and mental health level. We should pay timely attention to their psychological changes during peacekeeping missions, organize regular psychological screening, and implement psychological intervention targeting negative cognitive processing bias to reduce the occurrence of psychological problems.

## Author contributions

**Conceptualization:** Wen Jiang, Kaiwen Hou.

**Formal analysis:** Wen Jiang, Yiyi Zhang, Xu Dong, Kaiwen Hou.

**Investigation:** Wen Jiang, Yiyi Zhang, Xu Dong, Kaiwen Hou.

**Writing – original draft:** Wen Jiang.

**Writing – review & editing:** Kaiwen Hou.

## Supplementary Material


