# Supplementary material for: Observational study of the relationship between negative cognitive processing bias and mental health, sleep quality in the early and middle stages of peacekeeping mission
Source: Medicine (Baltimore). 2025 May 2;104(18):e42295. doi: 10.1097/MD.0000000000042295 (PMC12055113; doi:10.1097/MD.0000000000042295)
Supplement: Supplementary file 1 [file medi-104-e42295-s001.docx]

# Relationship between negative cognitive processing bias and mental health, sleep quality in the early and middle stages of peacekeeping mission

**Running title:** mental health of peacekeeping mission

Table S1 Demographic Characteristics of the participants (n=172)

| Variables | Grouping | n | % |
| --- | --- | --- | --- |
| Gender | Male | 172 | 100 |
|  | Female | 0 | 0 |
| Age (years) | 20−29 | 134 | 77.91 |
|  | 30−39 | 37 | 21.51 |
|  | 40−50 | 1 | 0.58 |
| Education level | Middle School | 79 | 45.93 |
|  | Junior college | 60 | 34.88 |
|  | Bachelor’s degree | 30 | 17.44 |
|  | Master’s degree or above | 3 | 1.74 |
| Marital status | Unmarried | 115 | 66.86 |
|  | Married | 56 | 32.56 |
|  | Divorced | 1 | 0.58 |
| Military age (years) | 1−9 | 146 | 84.88 |
|  | 10−19 | 21 | 12.21 |
|  | 20−30 | 5 | 2.91 |
| Military rank | Private | 142 | 82.56 |
|  | Officer | 30 | 17.44 |

Table S2. Comparison of SCL-90 results of participants in the early and middle stages of peacekeeping mission (±SD)


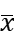


| Item | Early stage（n=172） | Middle stage（n=172） | t-value | P-value |
| --- | --- | --- | --- | --- |
| Somatization | 1.156±0.405 | 1.110±0.346 | 2.743 | 0.007 |
| Compulsive symptom | 1.165±0.424 | 1.103±0.338 | 2.908 | 0.004 |
| Interpersonal sensitivity | 1.083±0.358 | 1.056±0.326 | 2.053 | 0.042 |
| Depression | 1.079±0.351 | 1.047±0.316 | 2.760 | 0.006 |
| Anxiety | 1.075±0.351 | 1.053±0.314 | 1.745 | 0.083 |
| Hostility | 1.075±0.365 | 1.050±0.319 | 1.476 | 0.142 |
| Phobia | 1.045±0.324 | 1.034±0.308 | 1.173 | 0.242 |
| Paranoid | 1.068±0.354 | 1.039±0.313 | 2.080 | 0.039 |
| Psychotic symptom | 1.065±0.332 | 1.041±0.312 | 2.347 | 0.020 |
| Sleep and eating problems | 1.120±0.392 | 1.111±0.368 | 0.510 | 0.611 |
| Total score | 98.698±31.513 | 95.959±28.206 | 2.529 | 0.012 |

Table S3. Comparison of PSQI results of participants in the early and middle stages of peacekeeping mission (±SD)


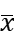


| Item | Early stage（n=172） | Middle stage（n=172） | t-value | P-value | |
| --- | --- | --- | --- | --- | --- |
| Sleep quality | 0.39±0.635 | 0.384±0.687 | 0.111 | | 0.912 |
| Sleep latency | 0.517±0.729 | 0.651±0.746 | -2.250 | | 0.026 |
| Sleep duration | 0.424±0.757 | 0.448±0.72 | -0.458 | | 0.648 |
| Sleep efficiency | 0.622±0.9 | 0.64±0.942 | -0.227 | | 0.820 |
| Sleep disorders | 0.483±0.577 | 0.494±0.577 | -0.277 | | 0.782 |
| Medication use to sleep | 0.017±0.229 | 0.035±0.263 | -1.742 | | 0.083 |
| Daytime sleepiness | 0.355±0.723 | 0.233±0.544 | 2.279 | | 0.024 |
| Total score | 2.808±3.274 | 2.884±3.114 | -0.387 | | 0.699 |

Table S4. Comparison of NCPBQ results of participants in the early and middle stages of peacekeeping mission (±SD)


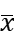


| Item | Early stage (n=172) | Middle stage (n=172) | t-value | P-value |
| --- | --- | --- | --- | --- |
| Negative attention bias | 6.913±2.480 | 6.529±2.441 | 1.925 | 0.056 |
| Negative memory bias | 7.890±2.921 | 7.203±2.729 | 2.963 | 0.003 |
| Rumination | 3.930±1.543 | 3.669±1.325 | 2.354 | 0.020 |
| Total score | 18.733±6.43 | 17.401±6.08 | 2.695 | 0.008 |
